# Microplastics in the Pathogenesis of Periodontal Diseases: A Narrative Review

**DOI:** 10.5334/aogh.4861

**Published:** 2025-10-03

**Authors:** Delfin Lovelina Francis, Saravanan Sampoornam Pape Reddy

**Affiliations:** 1 Saveetha Dental College and Hospital, SIMATS, Saveetha University, Chennai, India; 2 Department of Periodontology, 21 Corps Dental Unit, Bhopal, India

**Keywords:** dental, microplastics, oral health, periodontal, physiopathology

## Abstract

*Introduction:* Microplastics, plastic particles <5 mm in size, are a new class of environmental pollutants and have a role in systemic and oral health. Their implication in the pathogenesis of periodontal diseases has only recently been addressed.

*Objective:* This review will discuss recent evidence of the modes of action by which microplastics may be involved in the onset and development of periodontal diseases.

*Methods:* A systematic search of PubMed, Scopus, and Web of Science was performed up to May 2025 using keywords “microplastics,” “nanoplastics,” “oral health,” “periodontal disease,” “oxidative stress,” “dysbiosis,” “DNA damage response,” and “immune response” in the title, abstract, or keywords. According to PRISMA guidelines, 235 articles were retrieved, and 210 remained after duplicates were discarded. A total of 150 were removed after title/abstract screening. Sixty full-text articles were reviewed, and 30 were included in the qualitative synthesis.

*Results:* The existing evidence indicates that microplastics may induce periodontal pathology via several potential mechanisms, including (i) mechanical irritation of the surface of the gingival tissues, (ii) emission of toxic additives that cause oxidative stress, (iii) activation of DNA damage response (DDR) pathways, (iv) imbalance in the microbial community, and (v) immune regulation. These pathways intersect to enhance inflammation, tissue destruction, and dysbiosis, which culminate in the progression of periodontal disease.

*Conclusions:* It is suggested that microplastics are one of the potential epiphenomena of periodontal diseases. However, original experimental data are limited, especially with reference to immunological interactions. Future in-vitro and clinical investigations are urgently needed to confirm these mechanistic hypotheses and to foster preventive and therapeutic approaches.

## 1. Introduction

Gingivitis and periodontitis represent two of the most common chronic inflammatory diseases in humans, affecting an estimated 10% of the global population. They are marked by inflammation of the gingival tissues and progressive destruction of the tooth-supporting structures, namely the periodontal ligament and alveolar bone. Periodontal diseases have a multifactorial pathogenesis, which is a synergistic interaction of the host immune response with pathogenic microorganisms in the oral microbiota. The microbial insult is mainly due to pathogenic bacteria such as *Porphyromonas gingivalis (P. gingivalis), Fusobacterium nucleatum (F. nucleatum)*, and *Treponema denticola (T. denticola)*, which trigger inflammation, oxidative stress, and tissue destruction [1]. Recently, there has been a high interest in environmental pollutants, with a particular focus on microplastics as contributors to potential risk factors affecting human health, including oral health. Microplastics are plastic particles, typically defined as measuring <5 mm, which have entered the environment because of the breakdown of larger plastic items [2]. These particles are ubiquitous in food, water, air, and personal care products, contributing to accidental introduction into the human body through intake, inhalation, and dermal exposure. After entering the oral cavity, microplastics may directly contact gingival tissues, potentially causing both mechanical irritation and chemical toxicity. Although the impact of microplastics on different organ systems is still not fully understood, preliminary data show that these particles may contribute to the pathogenesis of periodontal disease through oxidative stress, inflammatory signaling, and microbial dysbiosis [3]. But the exact role microplastics play in the onset of periodontal disease has not yet been fully investigated.

Thus, the purpose of this narrative review is to critically explore the mechanisms by which microplastics may participate in the pathogenesis of periodontal diseases. In particular, this review highlights the effects of microplastic-driven oxidative stress, the activation of the DNA damage response (DDR), and the disturbance of microbial homeostasis in the periodontal environment.

## 2. Methodology

**Figure 1:**
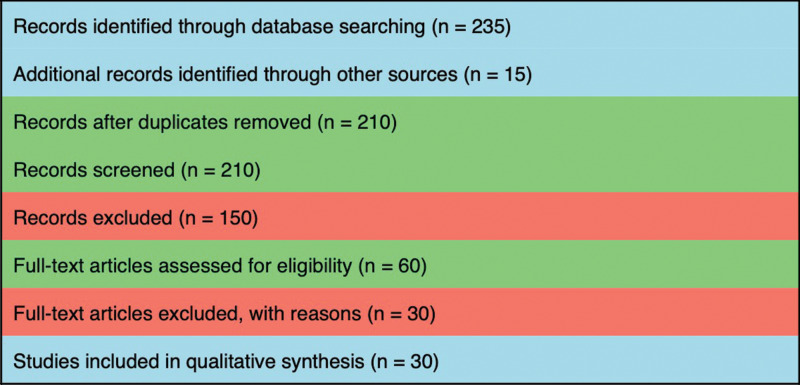
PRISMA flow diagram.

The review was based on PRISMA (preferred reporting items for systematic reviews and meta-analyses) criteria; it was not a systematic review but was an adapted narrative review from PRISMA [4]. A detailed search strategy was conducted in PubMed, Scopus, and Web of Science up to May 2025. The keywords used were “microplastics,” “nanoplastics,” “oral health,” “periodontal disease,” “oxidative stress,” “dysbiosis,” “DDR,” and “immune response.” Results were filtered using Boolean operators (AND, OR). The literature search and article selection process are illustrated in the PRISMA flow diagram (Figure 1). Only peer-reviewed English-language articles were included in the study. The inclusion criteria covered studies on microplastic exposure in biological systems with a potential relation to oral or periodontal health, including mechanistic in vitro studies, in vivo animal studies, and human observational data. Criteria for exclusion were studies not addressing microplastics, non-peer-reviewed publications, and conference abstracts. Titles and abstracts were independently screened by two authors, and full texts were reviewed for eligibility. Data were narratively synthesized on mechanistic pathways involving microplastics on potential contribution to their pathogenesis of periodontal disease.

## 3. Sources and Biological Uptake of Microplastics

Microplastics have become ubiquitous environmental pollutants, mostly derived from the degradation of large plastic litter, but also intentionally produced as small plastic particles used in various industrial and consumer applications [5]. These particles are ubiquitous in terrestrial, aquatic, and atmospheric environments, significantly impacting ecological and human health. In order to evaluate the direct influence of microplastics on the pathology of periodontal disease, we must be aware of their origin and routes of exposure to the body, specifically the mouth [6].

### 3.1 Primary and secondary sources of microplastics

Microplastics are categorized into two types: primary microplastics and secondary microplastics, depending on their origin. Primary microplastics are specifically manufactured tiny pieces of plastic with a specific purpose. These include microbeads that are present in personal care products such as facial scrubs, toothpaste, and cosmetics, as well as plastic pellets used in industrial applications. These particles can readily enter the human body when they are ingested or inhaled, as they are generally small [7]. Microbeads in toothpaste, for example, can stay in the oral cavity and may cause both irritation of the gingival tissues and exposure to toxic chemicals [8]. Secondary microplastics are generated through the breakdown of larger plastic items such as plastic bottles, bags, packaging materials, and synthetic fabric. Microplastics are produced when environmental weathering, such as UV radiation, mechanical abrasion, and chemical breakdown, gradually decreases the size of these plastic objects. In natural environments, secondary microplastics, which are the primary type found, are widely present in aquatic environments, soil, and suspended particulate matter. The human body is mainly exposed to these types of particles through the oral route via polluted water, food, and air. Once in the body, they can spread to other tissues, including the oral cavity [9].

### 3.2 Pathways of microplastic exposure to humans

Microplastics enter the human body through several pathways, with ingestion and inhalation being the most prevalent. These pathways are of particular relevance for periodontal disease, which is a consequence of the oral cavity being the gateway to both ingested and inhaled microplastics [10]. Microplastics are often ingested from contaminated food and water [11]. They have been found in marine organisms, including fish and shellfish. These tiny pieces of plastic have also been found in well water and bottled water, as well as other food. Once ingested, microplastics can traverse the gastrointestinal tract, and smaller particles can be absorbed into the bloodstream and transported to organs throughout the body [12].

Just like dust, microplastics are also found in the air, especially in industrial and urban areas. The majority of airborne microplastics are formed from the wear and tear of synthetic textiles, tires, and plastic products. After inhalation, these particles can remain in the upper respiratory tract and may potentially enter the oral cavity through mucociliary clearance or swallowing. The exposure is even increased with oral breathing through the nasal cavity and poses a risk for periodontal tissues, especially in people with pre-existing periodontal pathologies [13]. Microplastics have been less studied in terms of dermal exposure, but it is estimated that they can be absorbed into the skin while using personal care products containing microbeads. Microplastics in the oral cavity can then be inadvertently ingested via the oral mucous membrane during eating or drinking, resulting in microplastic exposure in the oral cavity [14]. The outlines of the most recent studies are presented in Table 1.

**Table 1:** Tabular representation of the recent most studies on microplastics.

AUTHOR (YEAR)	STUDY TYPE	MODEL/POPULATION	MAIN FINDINGS
Paul MB et al. [10]	Narrative review	Literature (oral uptake focus)	Comprehensive review of oral uptake, fate, and potential toxicity of micro-/nanoplastics (MNPs); highlights major knowledge gaps regarding exposure, biodistribution, and effects after oral uptake.
Mahmud F et al. [15]	Narrative review	In vitro and in vivo studies aggregated	Summarizes molecular/cellular effects of MNPs: induction of reactive oxygen species (ROS), inflammation, and senescence markers across cell lines and animal models; links MNP exposure to inflammatory and aging pathways.
Yang ZS et al. [16]	In vivo experimental	Mice, single oral exposure to MNPs	Demonstrated rapid absorption and distribution of NPs/MPs to blood and tissues (including adipose, nervous, and reproductive); shows size-dependent biodistribution and tissue invasion after oral exposure.
Zeng G. et al. [17]	Experimental (cellular + animal)	Caco-2 and mouse intestinal models	Polystyrene microplastics induced oxidative stress and intestinal barrier dysfunction via ROS → NF-κB / NLRP3 / IL-1β / MLCK pathway; antioxidants and inhibitors attenuated effects. (Mechanistic evidence for ROS → barrier dysfunction.)
“Impact of Micro- and Nanoplastics on Human Health” (review, 2020 PMC)	Narrative review	Broad literature synthesis	Reviews multiple organ/system effects of MNPs; summarizes mechanisms (oxidative stress, inflammation), and highlights the lack of human epidemiological evidence for many endpoints. Provides useful background for systemic consequences of oral exposure.
Polystyrene MPs — recent mechanistic papers (2024–2025)	Experimental studies	Cell and animal models	Multiple recent studies show PS-MPs promote oxidative stress, mitophagy alterations, and epithelial barrier dysfunction; antioxidant interventions reduce injury in some models (Collective mechanistic support for ROS → tissue dysfunction.)
Reviews connecting MNPs ↔ oral uptake & toxicity (2020–2023)	Reviews/perspective articles	Aggregated literature	Several recent reviews converge on the oral route as an important exposure path; emphasizes oral cavity as both a portal and a local exposure site; calls for targeted oral/gingival studies.
“Beyond microplastics — submicron plastics” (SpringerOpen, 2022)	Perspective/review	Literature	Discusses (sub)micron plastics, analytical challenges, and the relevance of small particles to tissue penetration—important context for particle size relevance to gingival penetration.
Polystyrene MP studies showing inflammatory signaling (various 2023–2024)	In vitro/in vivo mechanistic	Cell lines, rodents	Convergent evidence shows that PS microplastics trigger NF-κB signaling and NLRP3 inflammasome activation in epithelial and immune cells—a plausible route to chronic inflammation.
Systematic/narrative syntheses of MNP immunotoxicity (2022–2024)	Systematic and narrative reviews	Aggregated experimental studies	Reviews indicate consistent induction of oxidative stress and inflammatory mediators by MNPs, but note limited data on oral mucosa and periodontal immune responses specifically—a major evidence gap.

Apart from consumer and environmental sources, dental restorative materials might also be a potential source of microplastics in the oral cavity. Hybrid resin composites, resin-modified glass ionomers, and other polymerization-based restorative materials suffer wear from mastication on the one hand and from routine dental clinical treatment procedures on the other, with micro-/nanoplastic (MNP) debris released [18]. Additionally, when restorations are finished and/or polished, microplastic debris can be produced, which can either stay temporarily in the oral cavity or be ingested. These particles could act as local gingival irritants, further deteriorating inflammatory responses, or be swallowed, adding to systemic microplastic exposure [15]. As a result of the proximity to periodontal tissues, dental restorative-based microplastics are a previously unreported potential exposure pathway in dentistry and are clinically relevant [19].

### 3.3 Mechanisms of microplastic uptake in the oral cavity

After entering the oral cavity, microplastics interact with gingival tissues, and their mechanical and chemical effects may lead to periodontal disease. Microplastics enter the periodontium by several mechanisms (passive diffusion, cellular uptake, microplastic particles and gingival epithelial cell contact). Smaller microplastic particles, particularly those under 1 µm in size, can passively diffuse through the cellular membranes or be phagocytosed by immune cells such as macrophages and neutrophils [10]. These immune cells are recruited to the site of infection or inflammation and may also phagocytize microplastics as part of the immune response to foreign particles. Once microplastics enter the cells, they could cause cellular stress, initiate inflammatory pathways, or damage cellular functioning. In addition, microplastics could also pass into gingival epithelial cells to directly damage them and activate a variety of cellular stress responses such as oxidative stress [20].

Microplastics may also adhere to the surface of gingival epithelial cells, causing inflammatory responses in the localized tissues. These particles, when adhered to the oral mucosa, can result in mechanical irritation and damage or cause injury to the epithelial cells and stimulate the release of pro-inflammatory cytokines. The presence of microplastics could change the microenvironment of the periodontal tissues, leading to increased tissue destruction and inflammation [15]. If microplastics are ingested by oral tissues, they have the potential to migrate into the blood or lymphatic system. Previous studies have indicated that nanoparticles such as microplastics can cross the blood-brain barrier and accumulate in multiple systemic organs [21]. Thus, microplastics may potentially disseminate to the whole body through the oral route. Despite an increase in understanding, the exact processes by which microplastics enter periodontal tissues and contribute to disease progression are yet to be completely elucidated [16].

### 3.4 Chemical additives and toxicity of microplastics

Microplastics may act as both a physical irritant to the periodontal tissues and a chemical threat, as they can carry toxic additives. To improve properties, plastics are frequently combined with chemical compounds such as plasticizers, flame retardants, and stabilizers. Some of these chemicals, including bisphenol A (BPA), phthalates, and polybrominated diphenyl ethers (PBDEs), are endocrine disruptors and are capable of leaching from plastic materials upon degradation or contact with bodily fluids [13]. Upon the release of these additives from microplastics, harmful effects and consequences can be observed in the periodontal tissues and associated structures due to the induction of oxidative stress, genotoxicity, or inflammatory response mechanisms [22]. ROS produced from oxidative stress due to microplastics activates several critical signaling pathways, such as NF-κB, MAPK, and Nrf2, which lead to inflammation and tissue destruction in gingival tissues [17]. Chronic exposure to microplastics, along with the leaching of toxic chemicals, results in a sustained inflammatory state, which represents a feature of periodontal disease [23].

## 4. Role of Microplastics in Periodontal Disease Pathogenesis

**Figure 2:**
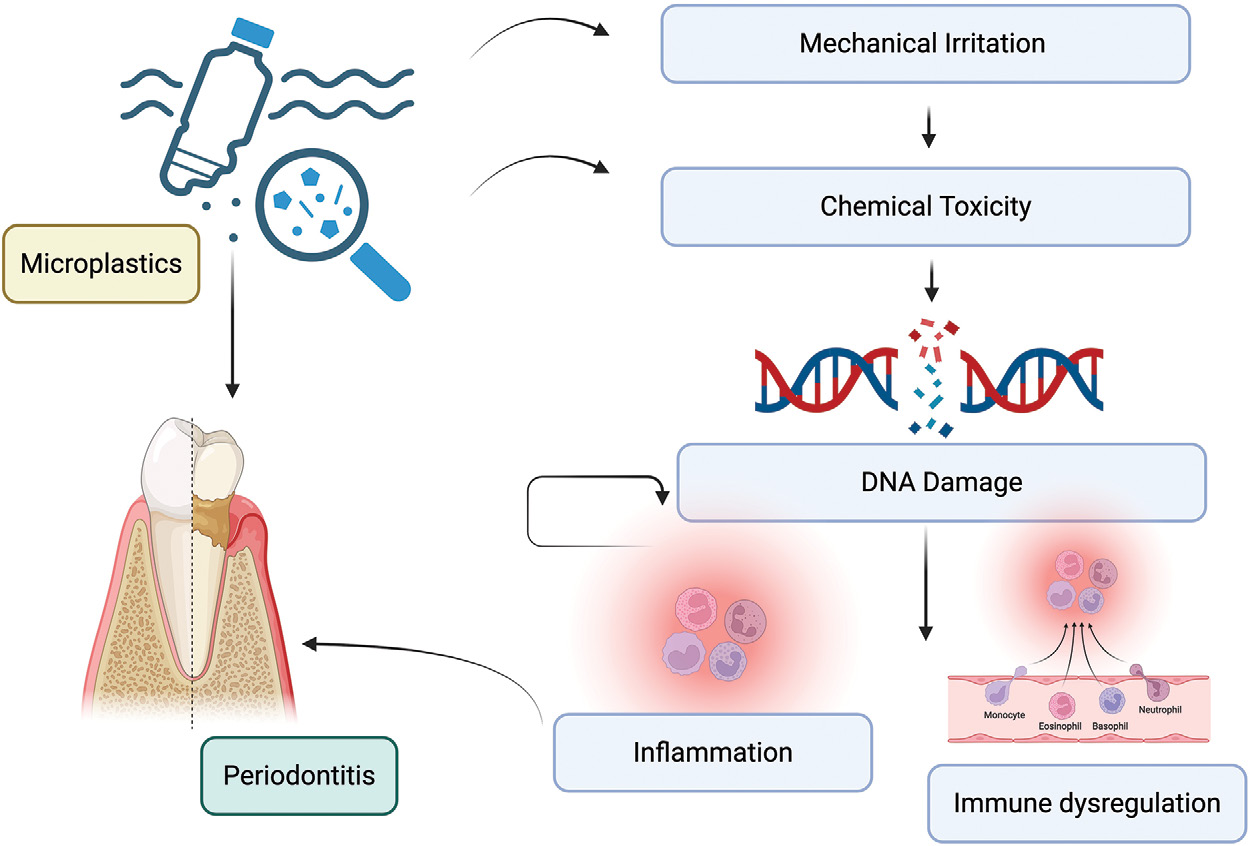
Mechanisms of action of microplastics on periodontium.

Periodontal diseases are chronic immuno-inflammatory conditions resulting from dysbiosis of subgingival microbiota and an increase of virulent pathogenic microorganisms in plaque, which exert adverse effects on homeostasis; however, periodontal pathogens, in particular, play a vital role in the progression of periodontal diseases, leading to the destruction of periodontal tissues, including the alveolar bone and the supporting structures of the teeth [24]. But more recent evidence indicates that environmental pollutants, such as microplastics, may play a potential role in the pathogenesis of periodontal disease via numerous pathways. Microplastics may alter periodontal disease through several mechanisms, including mechanical irritation, chemical toxicity, oxidative stress, immune dysregulation, and dysbiosis [25] (Figure 2).

### 4.1 Mechanical irritation and tissue damage

Mechanically, microplastics may induce periodontal disease by physical irritation of gingival tissues. Microplastics may act as local irritants when they interact with the gingival epithelium and are able to do so due to their physical properties, including size, shape, and surface texture. Microplastics, especially as fibers and fragments, might attach to the gingival surface with constant friction and microtrauma. After continued mechanical stimulation, the epithelial cells undergo damage, causing an inflammatory response leading to the breakdown in the continuity of the gingival tissue integrity. This results in the alteration of the epithelial barrier, rendering it more susceptible to additional microbial invasion [26]. Chronic irritation can lead to gingival recession as well as periodontitis and can provide a favorable environment for the replication of periodontal pathogens [27]. Mechanical irritation by microplastics can further trigger innate immune responses. Recruitment of neutrophils and macrophages to the area of irritation results in the secretion of pro-inflammatory cytokines and matrix-degrading enzymes, further worsening tissue destruction and disease progression [28].

### 4.2 Chemical toxicity and oxidative stress

Besides their physical characteristics, microplastics typically contain numerous toxic additives that can leach into surrounding tissues. These include plasticizers, stabilizers, and flame retardants, such as bisphenol A (BPA), phthalates, and polybrominated diphenyl ethers (PBDEs) [29]. These chemicals are recognized as endocrine disruptors and have been associated with the induction of oxidative stress [30]. Upon reaching periodontal tissues, microplastic particles can release these toxic substances, which yield ROS, causing oxidative damage in periodontal cells. Periods of oxidative stress play a principal role in the pathogenesis of periodontal diseases, as ROS can damage cellular macromolecules such as lipids, proteins, and DNA [31]. ROS-mediated injury initiates multiple intracellular signaling cascades related to inflammation, apoptosis, and DNA repair. Prolonged exposure to ROS in periodontal tissues can result in chronic inflammation, causing deterioration of periodontal health associated with microplastic exposure and impaired tissue repair capacity. Moreover, the expression of ROS may activate the NF-κB and MAPK pathways that cause the secretion of pro-inflammatory cytokines, including TNF-α, IL-6, and IL-1β. These cytokines lead to the destruction of the extracellular matrix and periodontal tissues [32].

### 4.3 Disruption of DNA repair mechanisms: activation of the DNA damage response pathway

Microplastics activate the DDR pathway via activation of oxidative stress/periodontal tissue-derived DNA damage. The DDR is an intricate signaling network that operates to sense and repair DNA damage to preserve genomic stability. The DDR plays a role in cell survival by allowing repair of DNA and preventing accumulated mutations under normal conditions. In the case of periodontal disease, however, the DDR pathway is dysregulated due to the persistent oxidative stress induced by microplastics. DNA lesions such as single-strand breaks (SSBs) and double-strand breaks (DSBs) occur when microplastics induce ROS generation [33]. These lesions, in turn, activate DDR sensors, including the MRN complex (MRE11, RAD50, NBS1), and kinases ATM (Ataxia Telangiectasia Mutated), and ATR (Ataxia Telangiectasia and Rad3-related). These kinases phosphorylate downstream effectors, including p53, CHK1, and CHK2, subsequently causing cell cycle arrest and DNA repair. This, consequently, affects periodontal tissue regenerative capacity, causing tissue loss and bone resorption. Beyond these maladaptive cellular responses, the activation of DDR pathways also upregulates inflammatory cytokines, contributing to a pro-inflammatory environment and further perpetuating periodontal tissue destruction.

### 4.4 Microbial dysbiosis and pathogen-microplastic interactions

The microbiome of the oral cavity reflects diversity in microbiota, and the changes in the homeostasis of organisms are associated with health and disease. Disruption of this microbial balance, known as dysbiosis, plays an important role in the pathogenesis of periodontal diseases. This is because microplastics may act as vectors in microbial dysbiosis, allowing bacteria to adhere to their surface and form biofilms [35]. This can lead to the expansion of pathogenic bacteria, such as *P. gingivalis, F. nucleatum,* and *T. denticola*, previously shown to propagate periodontal inflammation and tissue destruction. Microplastics can interact with periodontopathic bacteria in several ways. Molecularly, microplastics can act as a “colonisation substrate” for bacteria that form biofilms, thereby acting as a vector [36]. Second, microplastics are known to modulate the behavior of these pathogens by enhancing their virulence and resistance to host immune responses. For instance, *P. gingivalis* has been shown to attach to microplastic particles and develop biofilms, which shield the bacterium from immune surveillance by host organisms and even from antimicrobial therapies. Microplastics not only promote the growth of microorganisms but also cause a shift in oral microbiota composition, favoring the growth of pathogenic bacteria and/or inhibiting beneficial species. Such dysbiotic microbiota induce even more severe periodontitis by intensifying inflammation, raising oxidative stress, and upregulating pro-inflammatory cytokines and matrix-destroying enzymes [37].

### 4.5 Immune dysregulation and chronic inflammation

Microplastic exposure in periodontal tissues gives rise to a chronic inflammatory response that fuels the cycle of tissue destruction and attempt to regenerate. As stated above, microplastics induce oxidative stress and subsequently activate multiple immune signaling pathways such as NF-κB and MAPK. They also induce pro-inflammatory cytokines and matrix-degrading enzymes, promoting periodontal tissue degradation. Apart from ROS-mediated inflammation, microplastics also influence the functioning of immune cells. The continued exposure to microplastics may activate immune cells such as macrophages and neutrophils, which are recruited during the inflammatory process, causing hyper-responsiveness to inflammation [16]. The consequent overproduction of pro-inflammatory cytokines, including TNF-α and IL-1β, reinforces tissue damage through osteoclast activation. Moreover, chronic immune activation drives the accumulation of senescent cells that secrete further inflammatory mediators, perpetuating a vicious cycle of inflammation. This chronic pro-inflammatory environment is a signature of periodontal diseases and is responsible for the progressive destruction of the gingival tissues, periodontal ligament, and the alveolar bone. The dysregulated immune response could also damage the regenerative capacity of the periodontal tissues, where it could limit the natural healing processes of periodontal tissues [38].

### 4.6 Signaling pathways of oxidative stress

Exposure of periodontal tissues to microplastics leads to oxidative stress, which activates several important signaling pathways, including NF-κB signaling. As a pathway activated by ROS, NF-κB is a key regulatory mediator in inflammation. NF-κB mediates the oxidative stress induced by microplastics, which leads to persistent activation, chronic inflammation, and tissue degeneration [39]. The mitogen-activated protein kinase (MAPK) pathway is linked to the cellular response to stress and inflammation. ROS can also trigger the MAPK pathway, which induces the secretion of pro-inflammatory cytokines and contributes to periodontal tissue destruction [40]. The nuclear factor erythroid 2-related factor 2 (Nrf2) pathway is one of the most critical antioxidant defense pathways. Long-term exposure to microplastics might also interfere with the Nrf2 pathway, hampering the tissue’s ability to defend against oxidative injury [41].

## 5. The Role of Microplastics in Periodontal Disease Progression and Tissue Destruction

The interaction of microplastics, microbial dysbiosis, and the host immuno-inflammatory axis is crucial in the progression of periodontal disease. The persistent mechanical and chemical irritation, oxidative stress, and immune dysregulation caused by microplastic accumulation in the gingival tissues can provide a niche for periodontal pathogens to proliferate, whose ongoing presence and activity sustain the inflammatory process [42]. *P. gingivalis* and other periodontal pathogens not only promote the degradation of the periodontal tissues via matrix-degrading enzymes, such as collagenases and proteases, but also subvert host immune signaling to escape recognition and survive within the tissues. The biofilm matrix developed on microplastics also shields these pathogens from host immune responses and antimicrobial agents. The microbial burden intensifies, and inflammation continues, which leads to a rapid loss of the periodontal tissues, resulting in increased loss of the alveolar bone and tooth mobility [43]. Hence, exposure to microplastics worsens the essential mechanisms that are a precursor step toward periodontal disease, including the release of damage-associated inflammatory cytokines, which promote tissue destruction; immune evasion by pathogens, which enables them to survive and spread; and enhanced oxidative stress, which directly injures host tissues and facilitates the progression of disease. The cumulative impact of these mechanisms creates a detrimental cycle, reinforcing each process of microbial dysbiosis, inflammation, and tissue injury, leading to the advancement of periodontal disease [44]. Such studies pave the way for future research to fully elucidate the effects of microplastics on oral health and allow for the discovery of new therapeutic measures to minimize their role in periodontal disease. Utilizing advancements in antioxidant therapy, targeted gene delivery, and immunomodulation will enable overcoming the adverse effects of microplastics on periodontal tissues and facilitate tissue regeneration. It is certain that periodontal care in the future will need multidisciplinary collaboration and the utilization of advanced technologies to restore the overall equilibrium of the oral cavity and the environment amidst the ever increasing environmental pollutants [40].

## 6. Conclusion

The currently available data indicate that microplastics could play a potential role in the initiation and progression of periodontal diseases via mechanical stimulation, chemical toxicity, oxidative stress, microbial dysbiosis, and interference with the DNA damage repair machinery. Nevertheless, the possible immune consequences of microplastics on periodontal tissues are still mostly theoretical, and primary data remain scarce. Therefore, there is an urgent requirement for well-designed in vitro and causative studies to examine the cellular and molecular mechanisms by which microplastics engage gingival and periodontal immune responses. Such an evidence base will be critical to confirming current hypotheses and directing translational efforts to develop prevention and treatment strategies.
